# Comparative population genomics dissects the genetic basis of seven domestication traits in jujube

**DOI:** 10.1038/s41438-020-0312-6

**Published:** 2020-06-01

**Authors:** Mingxin Guo, Zhongren Zhang, Yanwei Cheng, Sunan Li, Peiyin Shao, Qiang Yu, Junjie Wang, Gan Xu, Xiaotian Zhang, Jiajia Liu, Linlin Hou, Hanxiao Liu, Xusheng Zhao

**Affiliations:** 1 College of Life Sciences, Luoyang Normal University, Luoyang, 471934 China; 2 Jujube Research Center, Luoyang Normal University, Luoyang, 471934 China; 3 Novogene Bioinformatics Institute, Beijing, 100083 China

**Keywords:** Plant domestication, Population genetics

## Abstract

Jujube (*Ziziphus jujuba* Mill.) is an important perennial fruit tree with a range of interesting horticultural traits. It was domesticated from wild jujube (*Ziziphus acidojujuba*), but the genomic variation dynamics and genetic changes underlying its horticultural traits during domestication are poorly understood. Here, we report a comprehensive genome variation map based on the resequencing of 350 accessions, including wild, semi-wild and cultivated jujube plants, at a >15× depth. Using the combination of a genome-wide association study (GWAS) and selective sweep analysis, we identified several candidate genes potentially involved in regulating seven domestication traits in jujube. For fruit shape and kernel shape, we integrated the GWAS approach with transcriptome profiling data, expression analysis and the transgenic validation of a candidate gene to identify a causal gene, *ZjFS3*, which encodes an ethylene-responsive transcription factor. Similarly, we identified a candidate gene for bearing-shoot length and the number of leaves per bearing shoot and two candidate genes for the seed-setting rate using GWAS. In the selective sweep analysis, we also discovered several putative genes for the presence of prickles on bearing shoots and the postharvest shelf life of fleshy fruits. This study outlines the genetic basis of jujube domestication and evolution and provides a rich genomic resource for mining other horticulturally important genes in jujube.

## Introduction

The Chinese jujube (*Ziziphus jujuba* Mill.) (2*n* = 2*x* = 24), a member of the Rhamnaceae family, is an important fruit tree with immense economic, ecological, and nutritional value. It has also traditionally been used as an herbal medicine. It originated in China and has been cultivated for over 7000 years^1^. In recent history, Chinese jujube has been dispersed to at least 47 countries on five continents^2^. It is now a major fruit crop with an estimated planting area of 3.25 million ha, and the total yield reached 8.52 million tons in China in 2017 (http://www.chyxx.com).

Cultivated jujube (*Z. jujuba*) was domesticated from wild jujube (*Ziziphus acidojujuba* C. Y. Cheng et M. J. Liu) through a long artificial selection process^1,3,4^ in which many important horticultural traits, such as fruit shape, kernel shape, bearing-shoot length, the number of leaves per bearing shoot, the presence of prickles on bearing shoots, the seed-setting rate and the postharvest shelf life of fleshy fruits, were altered. However, the genetic basis underlying these domestication traits remains largely unknown. Fruit shape or grain shape is a crucial trait that influences fruit or grain appearance in crops. Therefore, several genes responsible for fruit shape or grain shape have been identified in crops. For example, *GW7*^5^, *WTG1*^6^, and *GS9*^7^ regulate grain shape in rice; *OVATE*^8^ and *SUN*^9^ influence fruit shape in tomato; and *FUL1*^10^ determines fruit shape in cucumber.

Jujube has evolved a distinct self-shoot-pruning system that is very uncommon for perennial fruit crops. In jujube, the bearing shoot is the fruiting shoot. It is interesting that most bearing shoots are deciduous and typically drop before winter. This horticultural characteristic makes it easy to control tree size, is labor saving, and offers a unique model for deciphering shoot development in fruit crops^11^. The loss of prickles on bearing shoots is an obvious domestication trait. Most wild jujubes have sharp prickles on their bearing shoots, which help to protect them from herbivores. However, nearly all cultivated jujubes have evolved to exhibit smooth bearing shoots, which is convenient for farmers to harvest fruits. Previous studies revealed that the prickles were extensions or modifications of glandular trichomes^12,13^.

In addition to vegetative trait selection, the jujube reproductive system is another remarkable domestication trait that was modified during artificial selection. Like the vast majority of annual crops, wild jujubes are grown from seeds. However, cultivated jujubes are mainly clonally propagated because they produce few seeds, similar to other perennial fruit crops. It has been reported that over 75% of perennial fruit crops are clonally propagated^14^. Fruit softening is a major determinant of the length of the postharvest shelf life and commercial value. Therefore, delaying fruit softening is one of the major targets of fruit crop breeding. Fruit softening is associated with cell wall-modifying enzymes, including expansins, polygalacturonase, pectin methylesterase, pectate lyase, β-galactosidase, and cellulase^15–18^. The downregulation of polygalacturonase or pectate lyase expression leads to a longer postharvest shelf life in tomato^19,20^, strawberry^21,22^, and apple^23^.

Recently, based on next-generation DNA sequencing technologies, genome-wide association studies (GWASs) and selective sweep analysis have been performed to identify genes associated with horticulturally important traits in woody perennial fruit crops such as apple^24^, grape^25^, citrus^26,27^, pear^28^, and peach^29–31^. Due to the difficulty of jujube hybridization and a long juvenile phase, it is not easy to characterize the candidate genes underlying the horticultural traits of jujube using linkage mapping. Comparative population genomics provides a more suitable approach for identifying genes associated with domestication traits in jujube.

In this study, we performed deep genome resequencing of 350 wild and cultivated jujube accessions and analyzed their genomic variation dynamics during domestication. By integrating a GWAS approach with transcriptome profiling data, expression analysis and the transgenic validation of candidate genes, we identified a causal gene, *ZjFS3*, regulating fruit shape and kernel shape in jujube. Comparative population genomics also allowed us to identify the molecular mechanisms underlying several domestication traits, including bearing-shoot length, the number of leaves per bearing shoot, the presence of prickles on bearing shoots, the seed-setting rate and the postharvest shelf life of fleshy fruits. Furthermore, this study provides a rich genomic resource for mining other horticulturally important genes and achieving genomics-enabled molecular improvements in jujube.

## Results

### Genome resequencing, population structure, and genetic diversity

A total of 350 jujube accessions, including 41 wild jujube individuals, 23 semi-wild accessions and 286 jujube cultivars, were used in this study (Table S1). The resequencing of the 350 jujube accessions using the Illumina HiSeq PE150 platform generated a total of 2099.89 Gbp of sequences, with an average depth of more than 15× and coverage of 96.33% of the assembled genome (Table S2)^4^. After mapping against the jujube ‘Junzao’ reference genome^4^, we identified 10,355,825 single-nucleotide polymorphisms (SNPs) and 940,385 indels (≤5 bp) (Fig. S1 and Table S3). After filtering (coverage depth ≥ 6, MAF ≥ 0.05 and miss rate ≤ 0.1), a total of 1,688,146 high-quality SNPs were obtained for subsequent analysis. To validate the SNP calling results, three randomly selected SNPs were subjected to PCR amplification and Sanger sequencing in 55 accessions. The results indicated a high accuracy rate (96.2%) of our SNP calling results (Table S4).

**Fig. 1:**
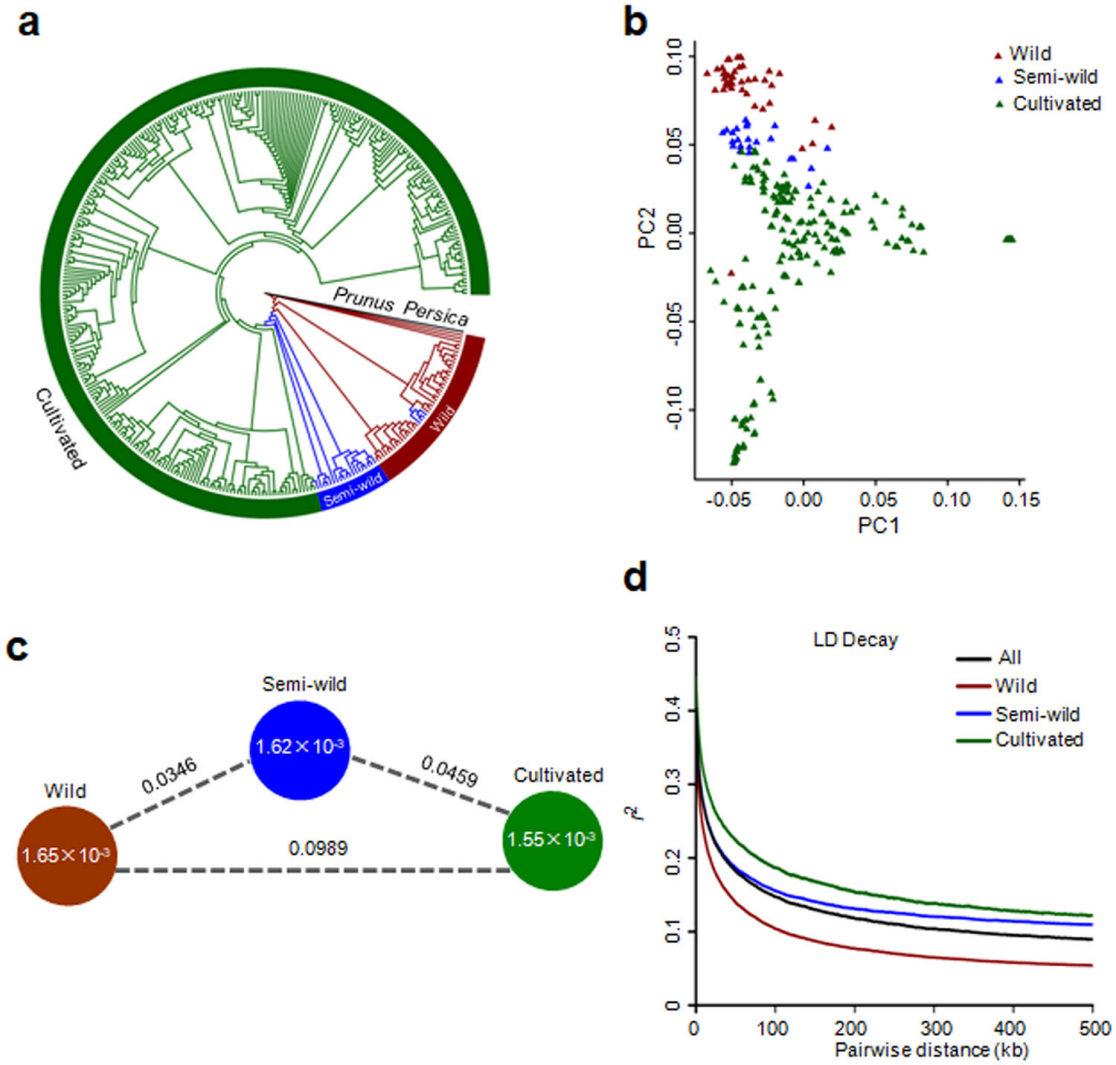
Population diversity and genetic differentiation analysis of wild, semi-wild and cultivated jujubes. **a** Phylogenetic tree of all accessions generated from 1,688,146 high-quality SNPs with *Prunus persica* as an outgroup. **b** PCA plots of the first two components for the 350 accessions. **c** Nucleotide diversity (*π*) and population differentiation (*F*_ST_) across the three groups. The value in each circle represents the nucleotide diversity of the group, and the value on each line indicates the population differentiation between the two groups. **d** Genome-wide decay of linkage disequilibrium (LD) in the three groups and all accessions

On the basis of phenotypic information, fruit size and other morphological traits, the 350 accessions were classified into three groups: wild, semi-wild and cultivated. To validate this classification, we explored the phylogenetic relationships among the 350 accessions by analyzing 1,688,146 high-quality SNPs using *Prunus Persica* as an outgroup. The resulting neighbor-joining tree supported the clustering of the three groups and illustrated that cultivated jujubes were domesticated from wild jujubes via semi-wild accession transition (Fig. 1a). Principal component analysis (PCA) illustrated a similar pattern to the phylogenetic tree in that the wild, semi-wild and cultivated accessions formed closely related clusters (Fig. 1b).

The nucleotide diversity (*π*) values in the wild, semi-wild and cultivated groups were 1.65 × 10^−3^, 1.62 × 10^−3^ and 1.55 × 10^−3^, respectively (Fig. 1c). The similar level of nucleotide diversity between jujube cultivars and their progenitor wild jujubes indicated a very weak bottleneck during artificial selection. However, through the comparison of *π* levels between wild accessions and cultivars, we found that the selection signals were significant in many genomic regions. The largest calculated *π*_w_/*π*_c_ (w: wild; c: cultivated) value was 11.52, which suggested that selection signals were strong in some genomic regions during jujube domestication and evolution (Fig. S2). The fixation index (*F*_ST_) value between the wild and cultivated groups was 0.0989 (Fig. 1c), which indicates moderate genetic differentiation between the two populations.

The extent of linkage disequilibrium (LD; indicated by *r*^2^) was measured as the genomic distance at which LD decreased to half of its maximum value. The LD extent in all the accessions was 34.2 kb (*r*^2^ = 0.20). The extent of LD in the wild, semi-wild and cultivated accessions was 20.2 kb (*r*^2^ = 0.19), 38.6 kb (*r*^2^ = 0.2) and 51.2 kb (*r*^2^ = 0.22), respectively (Fig. 1d). We also observed that the extent of LD decay on the 12 chromosomes was variable (Fig. S3).

### Selective sweep signals

To identify the potential selection signatures and genes underlying jujube domestication, three approaches, including the *π* ratio, *F*_ST_ and XP-CLR (the cross-population composite likelihood ratio)^32^, were implemented to compare the wild and cultivated groups. Although the wild and cultivated groups showed similar levels of nucleotide diversity, we identified a large number of potential genomic regions and selected genes using the three approaches (Fig. S2 and Tables S5, S6). On the basis of pairwise comparison between the wild and cultivated groups, we identified 2349, 1915, and 2003 candidate genes underlying artificial selection in the *π*_w_/*π*_c_, *F*_ST_ and XP-CLR analyses, respectively (Table S6). To confirm the selection signals and narrow down the potential candidate genes, we verified 1205 candidate genes by overlapping the *π*_w_/*π*_c_ and *F*_ST_ results, which indicated that more than 60% of the genes identified by *F*_ST_ could also be identified via the *π*_w_/*π*_c_ approach. Additionally, we overlapped the above three approaches and identified 196 candidates (Table S6). These genes may be involved in regulating domestication traits in jujube.

### Identification of fruit shape and kernel shape-related genes

**Fig. 2:**
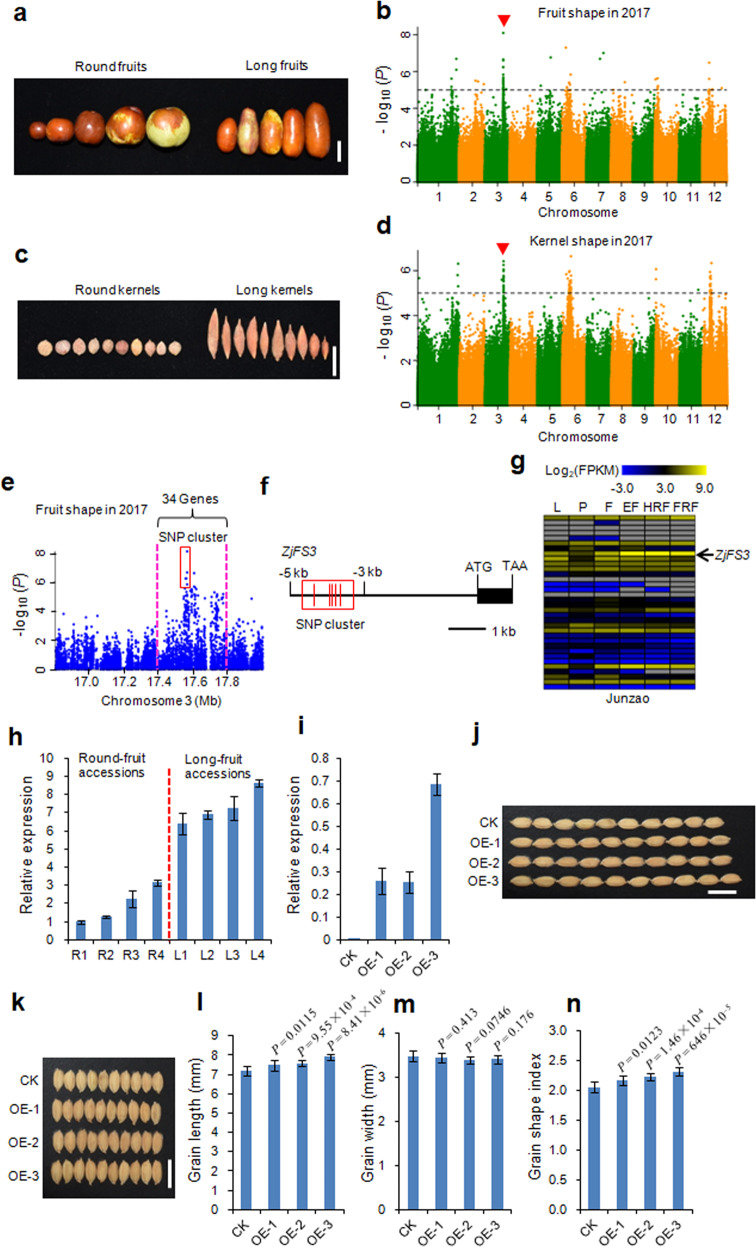
GWAS for fruit shape and kernel shape and the identification of the causal gene *ZjFS3*. **a** Phenotypes of jujube fruits. Bar = 2 cm. **b** Manhattan plots for fruit shape in 2017. Red arrowheads indicate the positions of the peaks identified in this study. Dashed lines represent significance thresholds (−log_10_*P* = 5). **c** Phenotypes of jujube kernels. Bar = 2 cm. **d** Manhattan plots for kernel shape in 2017. Red arrowheads indicate the positions of the peaks identified in this study. Dashed lines represent significance thresholds (−log_10_*P* = 5). **e** Local Manhattan plot surrounding the GWAS peak on chromosome 3. The red dashed lines indicate the candidate regions for the peaks. **f** Gene structure of *ZjFS3* and its nearby strongly associated SNPs for fruit shape (–log_10_*P* > 5). The corresponding SNP cluster is indicated by a red box. **g** Comparative tissue-specific transcript profiles of 34 genes in the GWAS peak in ‘Junzao’. The color scale represents FPKM-normalized log_2_-transformed counts. L, leaf; P, phloem; F, flower; EF, enlarged fruit; HRF, half-red fruit; FRF, fully red fruit. **h** Transcript levels of *ZjFS3* detected by qPCR in round fruits and long fruits at the fruit enlargement stage. The data shown are the mean values of three technical repeats with the SE. **i** Transcript levels of *ZjFS3* detected by qPCR in ‘Kongyu131’ (CK) and transgenic three overexpression (OE) lines. The data shown are the mean values of three technical repeats with the SE. **j**, **k** Grain morphology of CK and three OE lines, Bars = 1 cm. **l**–**n** Statistical data for grain length (**l**), grain width (**m**) and the grain shape index (**n**) in CK and three OE lines. Values are the means ± SD, *n* = 10. Differences between the CK and OE lines were analyzed with Student’s *t*-test

Most wild jujubes exhibit fruits and kernels with round or round-like shapes, while cultivated jujubes exhibit diverse fruit and kernel shapes. To dissect the genetic basis of fruit shape and kernel shape in jujube, we performed a GWAS using 350 accessions to identify candidate genes. The accessions used in this study showed diverse fruit shapes and kernel shapes, mainly including round and long shapes (Fig. 2a, c). Fruit shape indexes (FSIs—ratios of fruit length to fruit width) and kernel shape indexes (KSIs—ratios of kernel length to kernel width) were recorded in 2016 and 2017 and used as phenotypic data for the GWAS. The FSI and KSI values increased significantly during jujube domestication (Fig. S4).

We performed a GWAS using FSI and KSI as phenotypic data across two consecutive years (Figs. 2b, d and S5). A strong GWAS peak for fruit shape on chromosome 3 was identified in both the 2016 and 2017 data (Figs. 2b and S5), which overlapped with the peak for kernel shape (Figs. 2d and S5). Generally, round fruits harbor round or round-like kernels, and long fruits harbor long kernels based on our observations. Furthermore, we computed the correlation coefficient between FSI and KSI for the accessions used in the GWAS in two consecutive years, and these two traits showed a significant positive correlation (*r* = 0.417, *P* = 1.26 × 10^−15^, 2016 year; *r* = 0.35, *P* = 2.40 × 10^−11^, 2017 year). These results suggested that the same candidate gene regulates fruit shape and kernel shape.

We estimated a candidate region from 17.4 to 17.8 Mb harboring 34 putative genes (Fig. 2e). There were 30 SNPs (−log_10_*P* > 5) that showed a strong association with fruit shape in this candidate genomic region (Fig. 2e). Among these 30 SNPs, no nonsynonymous SNPs were identified. However, we found that a SNP cluster occurred in the upstream region (between −3 and −5 kb) of the start codon of *Zj.jz044531027*, encoding an ethylene-responsive transcription factor (Fig. 2e, f). We then analyzed the transcript levels of 34 putative genes in the candidate genomic region at different developmental stages in the ‘Junzao’ cultivar (Table S7). Notably, the *Zj.jz044531027* gene showed the highest expression levels at three of the fruit developmental stages (Fig. 2g and Table S7). Furthermore, we used two types of accessions (round-fruit and long-fruit accessions) to perform *Zj.jz044531027* expression analysis at the fruit-enlargement stage. The gene displayed higher expression in long-fruit accessions than in round-fruit accessions (Fig. 2h). Based on these combined results, we concluded that *Zj.jz044531027* was the key candidate gene regulating fruit shape and kernel shape in jujube and designated *Zj.jz044531027* as *ZjFS3* (*Fruit Shape Gene on Chromosome 3*).

To validate the biological functions of *ZjFS3*, we constructed the *Ubi::ZjFS3* binary plant transformation vector and generated rice overexpression lines. Among 45 independent lines, three transgenic overexpression lines (OE-1, OE-2, and OE-3) were selected for phenotypic analysis. The transcript level of *ZjFS3* in the above three OE lines was significantly upregulated (Fig. 2i). When we detected grain size and grain shape, we found that the grain lengths of the three OE lines were increased by 4.13%, 5.57% and 10.27%, respectively, compared to ‘Kongyu131’ (CK) (Fig. 2j, l). Grain width was not obviously changed (Fig. 2k, m). To evaluate grain shape, we determined the grain shape index (GSI —the ratio of grain length to grain width). The GSIs of all three OE lines were significantly increased (by 5.49%, 8.56% and 12.6% compared to CK, respectively) (Fig. 2n). These results confirm that *ZjFS3* is responsible for fruit shape and kernel shape in jujube.

### Increased bearing-shoot length and number of leaves per bearing shoot during jujube domestication

**Fig. 3:**
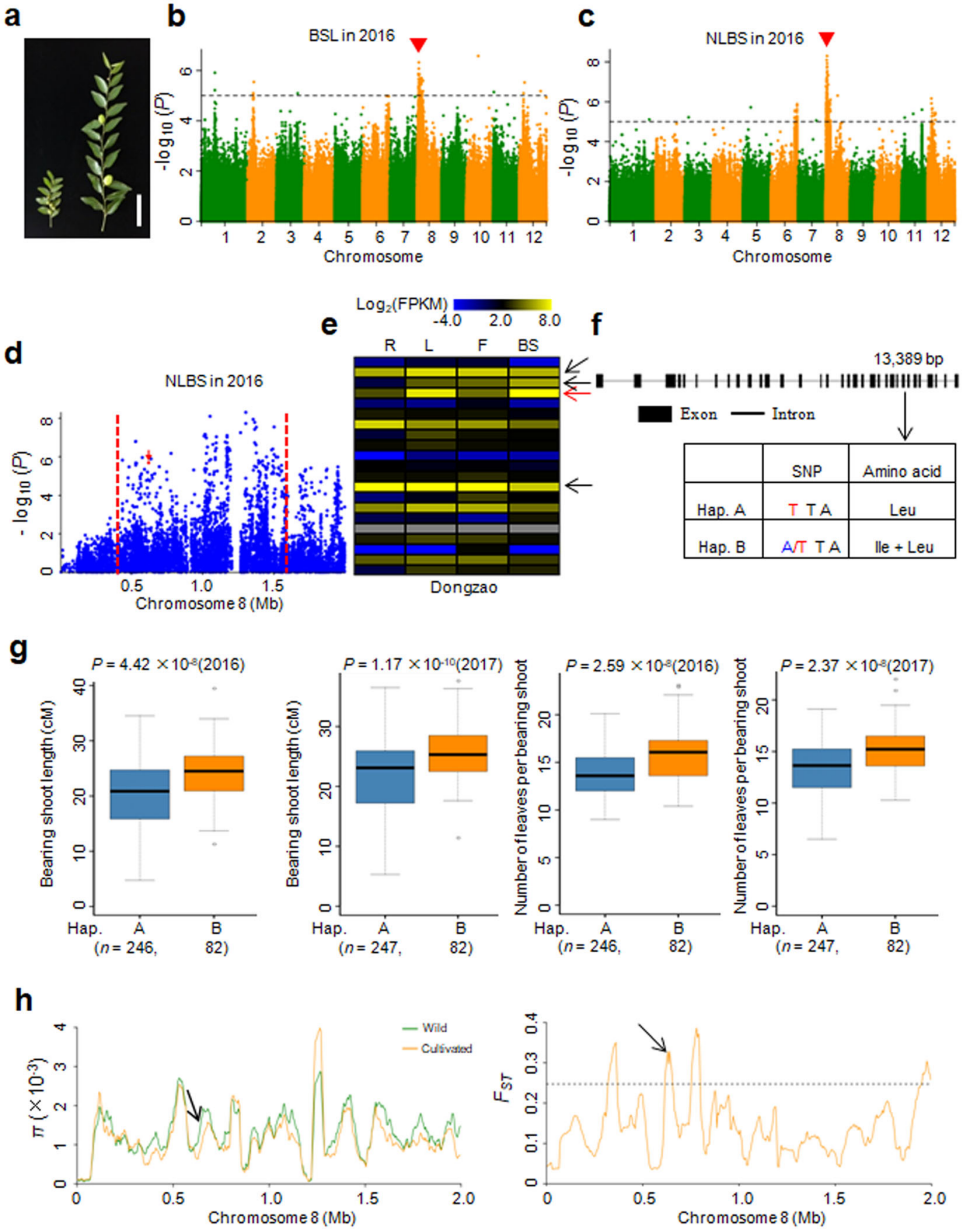
GWAS for BSL and NLBS and identification of the candidate gene *ZjNLBS1*. **a** Phenotypes of bearing shoots. Left, wild; Right, cultivated. Bar = 5 cm. **b**, **c** Manhattan plots for BSL (**b**) and NLBS (**c**). Red arrowheads indicate the positions of the peaks identified in this study. Dashed lines represent significance thresholds (−log_10_*P* = 5). BSL, bearing-shoot length; NLBS, number of leaves per bearing shoot. **d** Local Manhattan plot for NLBS surrounding the GWAS peak on chromosome 8. The red dashed lines indicate the candidate region for the peak. The arrow indicates the position of the nucleotide variation in *ZjNLBS1*. **e** Comparative tissue-specific transcript profiles of 21 genes with nonsynonymous SNPs in the GWAS peak in ‘Dongzao’. The color scale represents FPKM-normalized log_2_-transformed counts. R, root; L, leaf; F, flower; BS, bearing shoot. Arrows indicate that the four genes showed high expression levels in bearing shoots, and the red arrow indicates the *ZjNLBS1* gene. **f** Gene structure and DNA polymorphisms in *ZjNLBS1*. **g** Box plots for BSL and NLBS based on the two haplotypes of *ZjNLBS1* in 2016 and 2017. Bold central lines indicate the median, and the box limits represent the upper and lower quartiles. Whiskers extend to encompass data that fall within 1.5 times the interquartile range, and dots represent outliers. *n* indicates the number of accessions with the same haplotype. Significant differences were determined by the two-tailed Welch’s *t* test. **h** *ZjNLBS1* was embedded in domestication sweeps identified by the *π* ratio and *F*_ST_ analyses. The horizontal dashed line indicates the top 5% of genomic regions with the highest *F*_ST_ value

Bearing shoots are an important target for artificial selection in jujubes. The bearing-shoot length (BSL) and number of leaves per bearing shoot (NLBS) significantly increased during domestication (Figs. 3a and S4). To identify candidate genes associated with these two domestication traits, we performed a GWAS using BSL and NLBS as phenotypic data with all 350 accessions. We identified a strong GWAS peak on chromosome 8 for BSL (Fig. 3b), which overlapped with the peak for NLBS (Fig. 3c). Generally, a long bearing shoot harbors more leaves than a short bearing shoot (Fig. 3a). Furthermore, we computed the correlation coefficient between the BSLs and NLBSs of the accessions used in the GWAS. The results indicated that these two traits presented a significant positive correlation (*r* = 0.672, *P* = 3.68 × 10^−47^). These results also suggested that the same candidate gene is likely responsible for regulating BSL and NLBS.

For NLBS, a strong GWAS signal was mapped from 0.4 to 1.6 Mb on chromosome 8 (Fig. 3d). Within this region, there were 27 nonsynonymous SNPs that were significantly associated with NLBS (−log_10_ *P* ≥ 5). These 27 nonsynonymous SNPs were located within 21 putative genes (Table S8). We then analyzed the transcript levels of these 21 putative genes in the roots, leaves, flowers and bearing shoots of the ‘Dongzao’ cultivar (Fig. 3e). There were four genes that showed high expression levels in the bearing shoots (Fig. 3e and Table S8), and these four genes were annotated as transcription factor HBP-1b-like, transmembrane amino acid transporter, ferredoxin-dependent glutamate synthase and hypothetical protein genes. Based on the functional annotation of the orthologs in the model plants, we focused on *Zj.jz003639032* (designated *ZjNLBS1* for the NLBS trait), which encodes a ferredoxin-dependent glutamate synthase and showed the highest transcript level in the bearing shoots (Fig. 3e, red arrow). In rice, *ABC1/ES7* (*Os07g0658400*), an ortholog of *ZjNLBS1*, functions in nitrogen metabolism and plant growth^33,34^. Loss of function of *ABC1*/*ES7* led to a reduction in plant height.

There was one nonsynonymous T to A SNP located within *ZjNLBS1*. The allele at position 13,389 bp in exon 26 changed the amino acid from leucine to isoleucine and formed two types of haplotypes (Fig. 3f). Haplotype A harbored a TT homozygous allele, whereas haplotype B harbored an A/T heterozygous allele (Fig. 3f). The accessions carrying haplotype B exhibited longer bearing shoots and more leaves per bearing shoot than those with haplotype A (Fig. 3g). Moreover, *ZjNLBS1* was included in a selective sweep identified by both *π* and *F*_ST_ analyses (Fig. 3h and Table S6), which indicated that *ZjNLBS1*, underlying BSL and NLBS, was subjected to selection.

### Disappearance of prickles on bearing shoots during jujube domestication

**Fig. 4:**
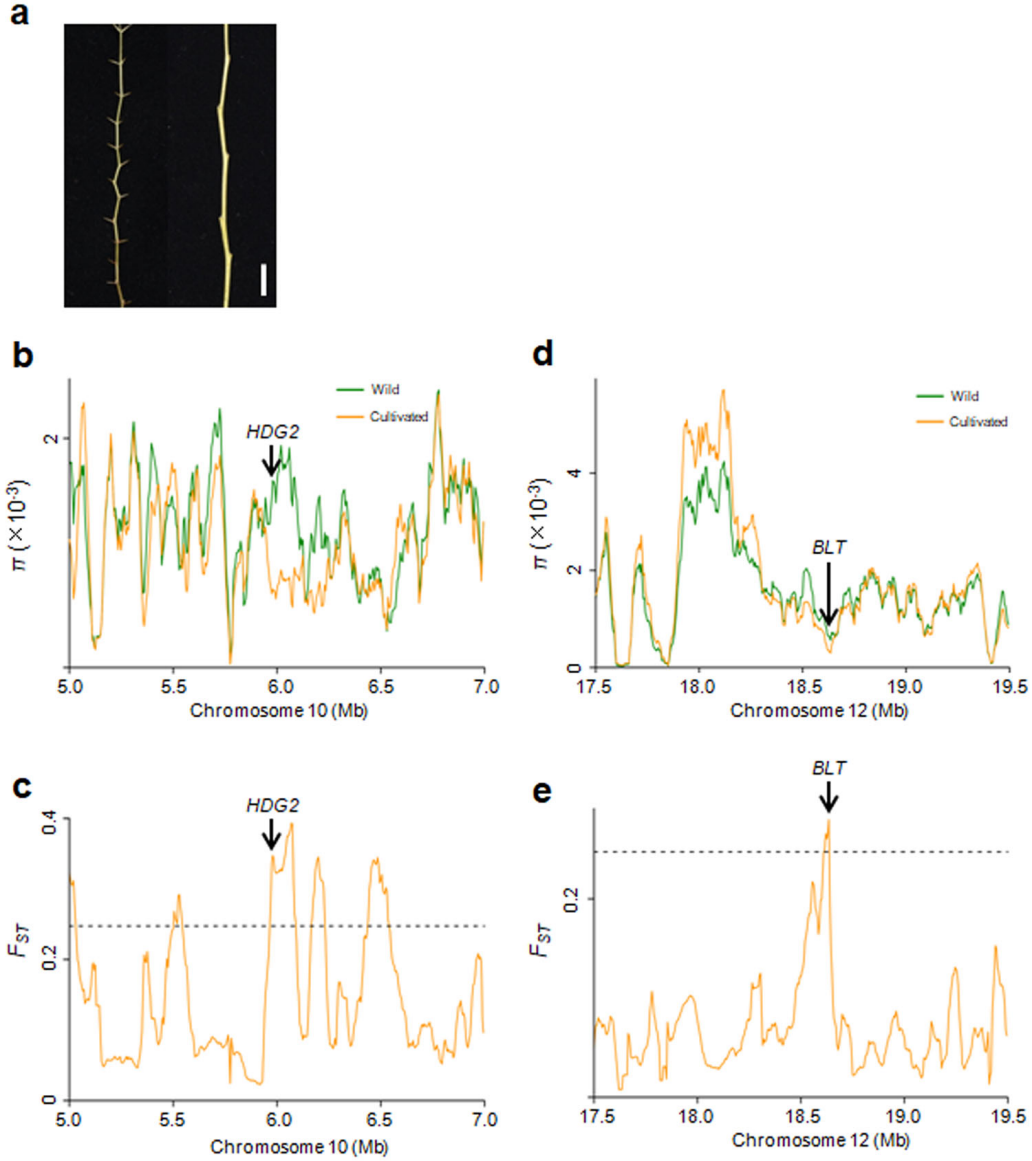
Domestication sweeps to identify the underlying genetic basis of the presence of prickles on bearing shoots. **a** Phenotypes of prickles on bearing shoots. Left, wild; Right, cultivated. Bar = 1 cm. **b**, **c** Distribution of *π* and *F*_ST_ values between the wild and cultivated groups spanning the region from 5.0 to 7.0 Mb on chromosome 10. The genomic location of *HDG2* is indicated. **d**, **e** Distribution of *π* and *F*_ST_ values between wild and cultivated groups spanning the region from 17.5 to 19.5 Mb on chromosome 12. The genomic location of *BLT* is indicated. The horizontal dashed lines indicate the top 5% of genomic regions with the highest *F*_ST_ values (**c**, **e**)

Most wild jujubes have sharp prickles on their bearing shoots (Fig. 4a). We investigated 33-wild accessions used in this study and found that 25 wild accessions exhibited prickles on their bearing shoots. Among 286 jujube cultivars, only one cultivar exhibited prickles on its bearing shoots. To elucidate the genetic basis underlying the selection for prickles on bearing shoots, we mined selective sweeps for genes that were potentially involved in regulating trichome development. We found that the candidate gene *Zj.jz044447010* on chromosome 10, which is the closest ortholog of *Arabidopsis HDG2* (*AT1G05230*), was under intensive human selection according to both *π* and *F*_ST_ analyses (Fig. 4b, c and Table S9). Similarly, another candidate gene, *Zj.jz040945037*, on chromosome 12, was also identified by both *π* and *F*_ST_ analyses (Fig. 4d, e and Table S9). It is the closest ortholog of *Arabidopsis BLT1* (*AT1G64690*). In *Arabidopsi*s, both *BLT1* and *HDG2* are involved in trichome development^35,36^. Therefore, the evolution of these two genes might have contributed to the disappearance of prickles on the bearing shoots of cultivated jujubes.

### Decrease in the seed-setting rate during jujube domestication

**Fig. 5:**
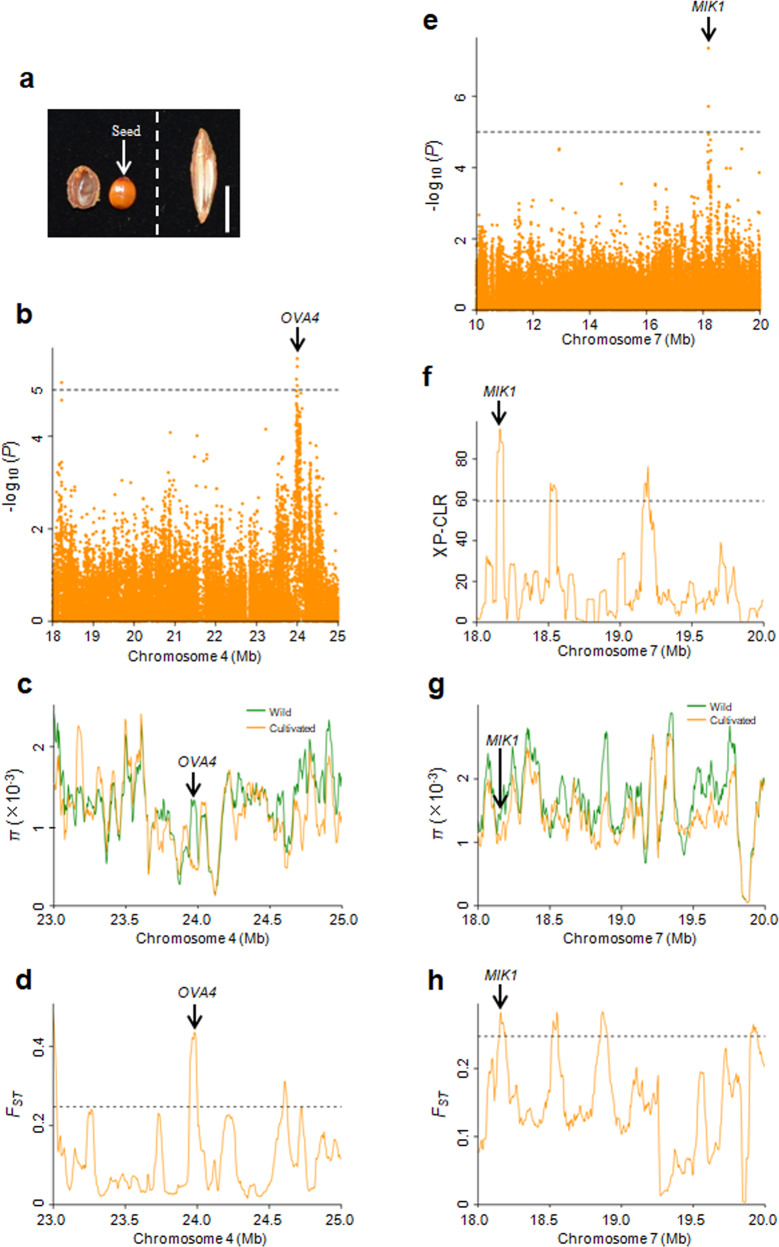
Selection targets for the seed-setting rate. **a** Phenotypes of longitudinal sections of kernels. Left, wild; Right, cultivated. Bar = 1 cm. **b** Manhattan plots of the GWAS results for the seed-setting rate on chromosome 4. Dashed lines represent significance thresholds (−log_10_*P* = 5). **c**, **d** Distribution of *π* and *F*_ST_ values between wild and cultivated groups spanning the region from 23.0 to 25.0 Mb on chromosome 4. The genomic location of *OVA4* is indicated. **e** Manhattan plots of the GWAS results for the seed-setting rate on chromosome 7. Dashed lines represent significance thresholds (−log_10_*P* = 5). **f**–**h** Distribution of XP-CLR, *π* and *F*_ST_ values between the wild and cultivated groups spanning the region from 18.0 to 20.0 Mb on chromosome 7. The genomic location of *MIK1* is indicated. The horizontal dashed lines indicate the top 5% of genomic regions with the highest *F*_ST_ (**d**, **h**) and XP-CLR (**f**) values

In addition to vegetative trait selection, a change in the reproductive system is a striking feature of the evolution and domestication of jujube (Fig. 5a). Cultivated jujubes were domesticated from wild ancestors through a selection process that resulted in the transition from sexual to asexual reproduction. The key factor influencing this transition was whether an embryo was formed. However, the molecular pathway that regulates embryogenesis in jujube remains largely unknown. To evaluate phenotypic variation in reproductive structures, we measured the seed-setting rate (kernels with full seeds/all detected kernels) of each accession. We found that the wild and semi-wild accessions exhibited much higher seed-setting rates than the cultivated accessions (Fig. S4). To identify candidate genes associated with the seed-setting rate, we performed a GWAS using 156 accessions, including 53 wild and semi-wild accessions and 103 cultivated accessions with low seed-setting rates (<10%).

Several GWAS loci were identified (Fig. S5), among which two overlapped with domestication sweeps (Fig. 5b–h). One GWAS locus on chromosome 4 overlapped with a domestication sweep supported by a high *π* ratio and *F*_ST_ values (Fig. 5b–d). This overlapping region includes a candidate gene, *ZjOVA4* (*Zj.jz006119092*), encoding a tryptophan-tRNA ligase, which is the closest ortholog of *Arabidopsis OVA4* (*AT2G25840*) (Table S9). In *Arabidopsis*, *OVA4* is required for ovule development, and the mutation of *OVA4* leads to ovule abortion^37^. Another GWAS locus on chromosome 7 was identified by XP-CLR, *π* ratio and *F*_ST_ analyses (Fig. 5e–h). There were 12 genes in this region of overlap (Table S9). One possible candidate gene, *ZjMIK1* (*Zj.jz007373151*), encodes a leucine-rich repeat receptor-like protein kinase and is the closest ortholog of *Arabidopsis MIK1* (*AT4G28650*). In *Arabidopsis*, MIK1 coupled with MIK2 and MDIS1 forms a cell-surface receptor heteromer on the pollen tube that responds to the female attractant LURE1^38^.

In addition to *ZjOVA4* and *ZjMIK1*, an ortholog (*ZjRAD51D*, *Zj.jz001293012*) of the *Arabidopsis* DNA repair protein *RAD51D* (*AT1G07745*) was found ~44.4 kb upstream from the significant SNP on chromosome 2, and another ortholog (*ZjYDA*, *Zj.jz034489027*) of the *Arabidopsis* MEKK subfamily member *YDA* (*AT1G63700*) was found ~37.7 kb upstream from the significant SNP on chromosome 4 (Fig. S5 and Table S9). *AtRAD51D* regulates homologous recombination^39^.

*AtYDA* promotes extraembryonic cell fates in the basal lineage, and a loss of function *yda* mutant shows embryo lethality^40^. These candidate genes may be the cause of significantly reduced seed-setting rates in cultivated jujubes compared to wild individuals.

### Prolonging the postharvest shelf life of fleshy fruits during jujube domestication

**Fig. 6:**
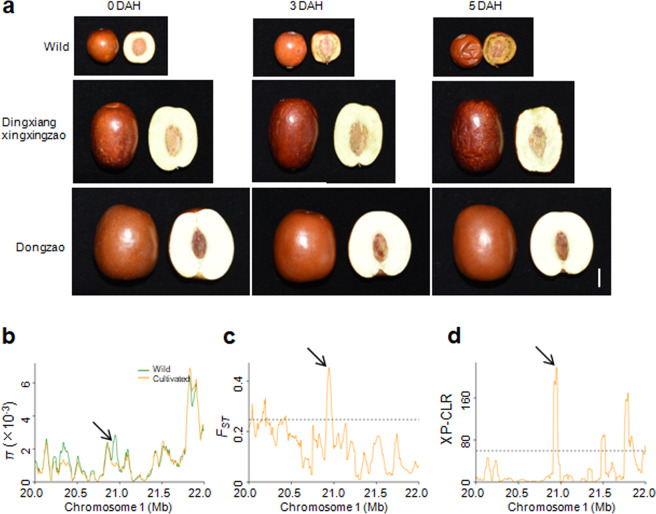
Domestication sweeps to identify the underlying genetic basis for the postharvest shelf life of fleshy fruits. **a** Phenotypes of three accessions’ fruits (one wild accession, ‘Dingxiangxingxingzao’ and ‘Dongzao’) after harvesting. The fully red fruits were harvested and photographed at 0, 3, and 5 days after harvesting. Bar = 1 cm. DAH, days after harvesting. **b**–**d** Distribution of *π*, *F*_ST_ and XP-CLR values between wild and cultivated groups spanning the region from 20.0 to 22.0 Mb on chromosome 1. The arrows indicate the genomic location of *Zj.jz044553003*. The horizontal dashed lines indicate the top 5% of genomic regions with the highest *F*_ST_ (**c**) and XP-CLR (**d**) values

Previous studies revealed that fruit softening is associated with cell wall-modifying enzymes including polygalacturonase, pectin methylesterase, pectate lyase, β-galactosidase, cellulase and expansins^15–18^. When we harvested and stored the mature fruits of jujubes, we found that the fruits of the wild accessions softened more easily than those of the cultivated species. Specifically, we chose one wild accession, one dry cultivar (‘Dingxiangxingxingzao’) and one fresh cultivar (‘Dongzao’) to compare the extent of fruit softening after harvesting. The results showed that the wild accession started to soften three days after harvesting and softened completely by five days (Fig. 6a). In contrast, the fruits of the ‘Dingxiangxingxingzao’ and ‘Dongzao’ cultivars showed no obvious phenotypic change at three days after harvesting, and ‘Dingxiangxingxingzao’ fruits showed only mild softening at five days, while the fruits of ‘Dongzao’ showed no change at five days (Fig. 6a).

To elucidate the genetic basis underlying selection for fruit softening, we mined selective sweeps for genes that were potentially involved in cell wall modification. We found a region on chromosome 1 that was under intensive artificial selection according to *π* ratio, *F*_ST_ and XP-CLR analyses (Fig. 6b–d). This region harbored a gene (*Zj.jz044553003*) encoding the key cell wall modifying enzyme polygalacturonase. Furthermore, by overlapping the *π* ratio and *F*_ST_ analyses, we found that genes encoding polygalacturonase, pectinesterase and expansin were included in the domestication sweeps (Table S10). Therefore, these genes may contribute to the significantly prolonged postharvest shelf life of fleshy fruits in cultivated jujubes compared to wild species.

## Discussion

To date, two reference jujube genomes, for ‘Dongzao’ and ‘Junzao’, have been released (in 2014 and 2016 years^4,11^, respectively). However, the genetic basis underlying horticulturally important traits in jujube remains largely unknown. In this study, we attempted to dissect the molecular mechanisms of seven domestication traits in jujube based on resequencing 350 accessions, including wild and cultivated individuals.

For fruit shape and kernel shape, we characterized a causal gene, *ZjFS3*, regulating fruit shape and kernel shape in jujube. The overexpression of *ZjFS3* in rice led to increased grain length but did not influence grain width (Fig. 2j–m). Therefore, we conclude that *ZjFS3* regulates fruit shape by modulating fruit length. To support this hypothesis, we performed a GWAS using fruit length and fruit width as the phenotypic data. For fruit length, a GWAS signal was identified on chromosome 3 (Fig. S5), which overlapped with the GWAS locus for fruit shape. However, for fruit width, we did not identify a GWAS signal on chromosome 3 (Fig. S5). Therefore, these results led us to infer that *ZjFS3* regulates fruit shape by modulating fruit length in jujube. *GS9* regulates grain shape by simultaneously modulating grain length and grain width in rice. The knockout of *GS9* led to an increased grain length and decreased grain width^7^. Similarly, *SUN* controls tomato shape by increasing cell division in the longitudinal direction and decreasing cell division in the transverse direction of the fruit^9^. Thus, *ZjFS3* provides a new mechanism regulating fruit shape compared to *GS9*^7^ and *SUN*^9^.

Bearing shoots are not only a key domestication target in artificial selection but also a trait of scientific interest. In jujube, leaves, flowers and fruits originate on the bearing shoot. It germinates from the fruiting section in spring and is deciduous, typically dropping before winter. This makes jujube distinct from other perennial fruit crops but similar to annual crops in a sense. In this study, we identified a candidate gene, *ZjNLBS1*, that potentially regulates BSL and NLBS. Its ortholog, *ABC1/ES7*, functions in plant height in rice^33,34^. Based on phenotypic observations and biological functions, we propose that bearing-shoot length in jujube is comparable to plant height in annual crops. Thus, we consider *ZjNLBS1* to be an important candidate gene for future research.

The change in the reproductive system is a notable domestication event that occurred in jujube. By overlapping GWAS and selective sweep analysis, we identified two candidate genes, *ZjOVA4* and *ZjMIK1*. Their orthologs in *Arabidopsis* regulate ovule development and the male detection of female attraction signals, respectively^37,38^. Previous work showed that many factors result in low seed-setting rates in modern cultivated jujubes, including a low pollen germination rate, pollen tube growth arrest, and embryo abortion after fertilization^41–43^. Moreover, the embryo abortion stage differs among jujube cultivars. Several accessions show embryo abortion several days after fertilization, whereas other accessions exhibit normal embryo development until the spherical embryo stage^41,43^. Thus, in different jujube cultivars, there may be different mechanisms underlying low seed-setting rates.

In this study, we identified a set of candidate genes associated with domestication traits in jujube via GWAS and selective sweep analysis. However, fully elucidating the contribution of candidates will require the overexpression or knockout of these candidates in jujube. Thus far, genome editing has been used to validate the functions of candidate genes and improve important traits in horticultural crops^44,45^. However, a series of factors make this transgenic work challenging, such as the difficulty of *Agrobacterium*-mediated transformation and the long generation time of jujubes. The genomic resequencing data will also be valuable for further research on the metabolism and quality traits of jujube fruits.

During jujube domestication, phenotypic change was a successive process. In addition to wild and cultivated accessions, there are several intermediate-type accessions whose organs are larger than those of wild jujube but smaller than those of cultivated jujube^1^. Previous publications have classified these intermediate-type accessions as semi-wild or semi-cultivated^4,46^. In this study, we also collected 23 accessions belonging to the semi-wild group. We focused on dissecting the genetic basis of seven domestication traits through GWAS and selective sweep analysis. For the latter analysis, we chose to examine the wild vs. cultivated groups for the identification of candidate domestication genes since these two groups exhibit distinct morphological variations. In this study, we did not provide insights into the dynamic genetic basis during the artificial selection process from the wild to semi-wild and semi-wild to cultivated groups. The genomic variation dynamics in the possible two-step domestication process and the evolutionary history of jujube will be examined in our future work.

## Materials and methods

### Sample collection and agronomic evaluation

A total of 350 accessions were sampled from two germplasm repositories. The majority of the jujube cultivars (285/286), partial semi-wild accessions (8/23) and one wild accession were sampled from the National Jujube Germplasm Repository of China, Pomology Institute, Shanxi Academy of Agricultural Sciences (Taigu County, Shanxi Province). The majority of the wild accessions (40/41), over half of the semi-wild accessions (15/23) and one jujube cultivar were sampled in the Tuokexun Jujube Germplasm Repository (Tuokexun County, Xinjiang Uygur Autonomous Region, China). During the growing period, all accessions in the two germplasm repositories were managed according to standard procedures.

For GWAS, five agronomic traits, including fruit shape, kernel shape, bearing-shoot length, the number of leaves per bearing shoot and the seed-setting rate, were investigated and evaluated in 2016 and 2017. The fruit shape index (FSI) was determined as the ratio of fruit length to fruit width. Similarly, the kernel shape index (KSI) was determined as the ratio of kernel length to kernel width. The seed-setting rate is equal to the number of kernels with full seeds/total number of detected kernels. These traits were evaluated and characterized based on the previously published ‘*Descriptors and Data Standard for Jujube* (*Ziziphus jujuba* Mill.)’^47^. Briefly, the seed-setting rate was evaluated using over 30 fruits, and the other traits were all evaluated using ten replicates. Fruit length, fruit width, kernel length and kernel width were determined by using Vernier calipers. Bearing-shoot length was determined with a common ruler. When evaluating the seed-setting rate, the kernels were crosscut, and the kernels with full seeds were counted. The average values of these traits were used for GWAS. To evaluate the extent of the postharvest shelf life of fleshy fruits, fully red fruits with a hard texture were harvested and stored at room temperature. The fruits were photographed at 0, 3, and 5 days after harvesting.

### Genome sequencing

Total genomic DNA (500 ng per sample) was extracted from the young leaves of 350 jujube accessions. Sequencing libraries were generated using the Truseq Nano DNA HT Sample Preparation Kit (Illumina USA) following the manufacturer’s recommendations. These libraries were sequenced on the Illumina HiSeq X ten platform to obtain 150 bp paired-end reads. Finally, we obtained 2099.89 Gbp of high-quality paired-end reads.

### Variation calling

The high-quality paired-end reads were aligned against the jujube reference genome^4^ using BWA (Burrows-Wheeler Aligner) (Version: 0.7.8)^48^ with the command ‘mem -t 4 -k 32 -M’. Then, SAMtools software^48^ was used to convert the alignment results to BAM files, and duplicate reads from PCR amplification were removed by using SAMtools^48^. For SNP calling, we used a Bayesian approach as implemented in the package SAMtools^48^. For each accession, we calculated the genotype likelihood and allele frequencies from the reads at each genomic location with a Bayesian approach. We then used the ‘mpileup’ command to identify SNPs with the parameters ‘-q 1 -C 50 -S -D -m 2 -F 0.002 -u’. The method for indel calling was similar to that for SNP calling, and only indels of ≤5 bp were taken into account.

### Phylogenetic tree and principal component analysis

A total of 1,688,146 high-quality SNPs were used for phylogenetic and population structure analyses. Via the neighbor-joining method, a phylogenetic tree including 350 accessions was constructed using the software TreeBest v1.9.2^49^ with 1000 bootstrap replicates, and *Prunus persica* was used as the outgroup. In addition, we performed PCA to evaluate genetic structure using the software GCTA^50^, and the Tracy-Widom test was employed to determine the significance level of the eigenvector.

### Linkage disequilibrium analysis

Haploview^51^ was used to calculate the squared correlation coefficient (*r*^2^) between pairwise SNPs for the wild, semi-wild and cultivated accessions. The parameters of the program were set as follows: ‘-n -dprime -minMAF 0.05’. The squared correlation coefficient (*r*^2^) values were analyzed in 500-kb windows across the whole genome.

### Selective sweep analyses

To identify genome-wide selective sweeps, three approaches, including the genetic diversity (*π*), fixation index (*F*_ST_) and the likelihood methods (XP-CLR)^32^, were adopted. We scanned the genome in 50-kb sliding windows with a 5-kb step size and calculated the genome-wide distribution of genetic diversity (*π*) and the fixation index (*F*_ST_) in wild and cultivated accessions by using vcftools^52^. Windows in the top 5% of *π*_wild_/π_cultivated_ values and *F*_ST_ scores were considered candidate regions for domestication sweeps.

For XP-CLR analysis, a 0.05-cM sliding window with 100-bp steps across the whole genome was used for scanning. We fixed the maximum number of SNPs assayed in each window to 200, and the command line was as follows: XP-CLR − c freqInput output File −w1 gWin(Morgan) snpWin gridSize(bp) chrN. Finally, we calculated the mean likelihood score in 50-kb sliding windows with a step size of 5 kb across the genome. The windows with the top 5% of XP-CLR values were considered selected regions.

### Genome-wide association study (GWAS)

We used the GEMMA^53^ (genome-wide efficient mixed-model association) software package to conduct association analysis. For the mixed-linear-model (MLM) analysis, we used the equation

$$y = X\alpha + S\beta + K\mu + e$$

In this equation, *y*, *X*, *S*, and *K* represent the phenotype, genotype, structure matrix and relative kinship matrix, respectively. *X*α and *S*β represent fixed effects, and *Kμ* and *e* represent random effects. For population structure correction, the top three PCs were used to build up the *S* matrix. The *K* matrix was built using a matrix of simple matching coefficients. The GWAS thresholds of all tested traits were evaluated with the formula *P* = 1/*n* (*n*, the effective number of independent SNPs, which was calculated using Genetic type 1 Error Calculator (GEC) software^54^). Finally, the threshold was estimated to be ~1.0 × 10^−5^.

### Transcriptome analysis and qPCR

RNA-seq data from two cultivars, ‘Junzao’ and ‘Dongzao’, have been reported previously^4,11^. Raw data were downloaded from NCBI with accession numbers SRX1518646, SRX1518647, SRX1518648, SRX1518650, SRX1518651, SRX1518652, SRX1518653, SRX1518654, SRX1518655, and SRX1518656 for ‘Junzao’; SRX691529, SRX691531, SRX691533, and SRX691539 for ‘Dongzao’. Then, the raw data were filtered and trimmed to yield clean reads, and these high-quality reads were mapped to the reference genomes^4^ with HISAT^55^. The expression level (FPKM value) of each protein-coding gene was calculated by Cufflinks (https://github.com/cole-trapnell-lab/cufflinks).

For qPCR, total RNA was extracted using a TaKaRa MiniBEST Plant RNA Extraction Kit and reverse transcribed with a PrimeScript II 1st Strand cDNA Synthesis Kit (TaKaRa). Quantitative real-time PCR was performed in triplicate with TB Green^TM^ Premix Ex Taq^TM^ II (TaKaRa). *ZjActin* (GenBank accession number, KT381859) and *OsActin* (*Os03g0718100*) were used as endogenous genes. The relative quantification method (2^−ΔΔCT^) was used to evaluate quantitative variation between the examined replicates^56^. The primers used for qPCR are listed in Table S11.

### Gene cloning and transgenic experiment

For transgenic validation, the full-length coding sequence of the *ZjFS3* gene was amplified using jujube fruit cDNA. The amplified product was further ligated into the pZH2Bi vector driven by the *ubiquitin* promoter. The rice variety ‘Kongyu131’ (*japonica*) was used as the receptor for transformation by *Agrobacterium*. Plant transformation was conducted as described previously^57^. The primers used for vector construction are listed in Table S11.

## Supplementary information

Figures S1-S5

Table S1

Table S2

Table S3

Table S4

Table S5

Table S6

Table S7

Table S8

Table S9

Table S10

Table S11

## Data Availability

All the genomic sequence datasets have been deposited in the NCBI Sequence Read Archive under accession number PRJNA560664.
