# Supplementary material for: Comparative population genomics dissects the genetic basis of seven domestication traits in jujube
Source: Hortic Res. 2020 Jun 1;7:89. doi: 10.1038/s41438-020-0312-6 (PMC7261808; doi:10.1038/s41438-020-0312-6)
Supplement: Supplementary file 1 — Figures S1-S5 [file 41438_2020_312_MOESM1_ESM.docx]

**Fig. S1 A Circos image representing genome-wide variations identified across 350 jujube accessions. 1** 12 pseudo-chromosomes in different colors with scale of Mb genome. **2** Sequence coverage. The range of the coverage plot axis is 0 to 16,000. **3** The number of SNP in genome.The range of the SNP axis is 0 to 42,000. **4** The number of indels (light purple, insertions; dark purple, deletions) in genome. The range of the indel axis is 0 to 2,300.


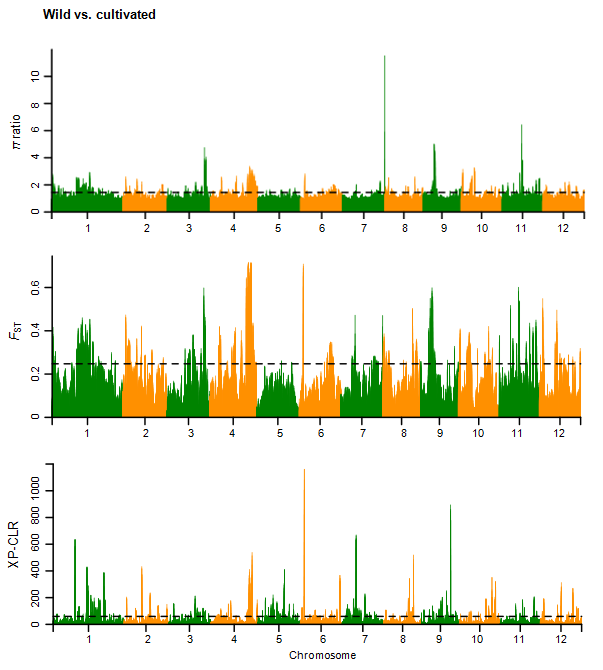


**Fig. S2 Genome-wide distribution of selective sweeps identified by *π* ratio, population differentiation (*F*_ST_) and XP-CLR analyses.** Gray dotted lines represents the top 5% threshold for *π* ratio, *F*_ST_ and XP-CLR values.


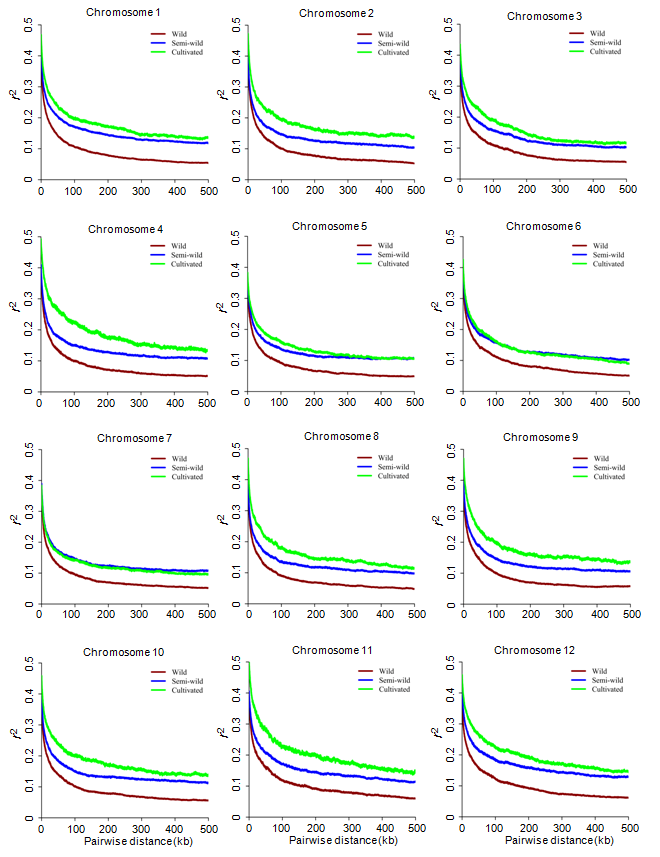


**Fig. S3 Decay of linkage disequilibrium (LD) among three groups in each chromosome.**


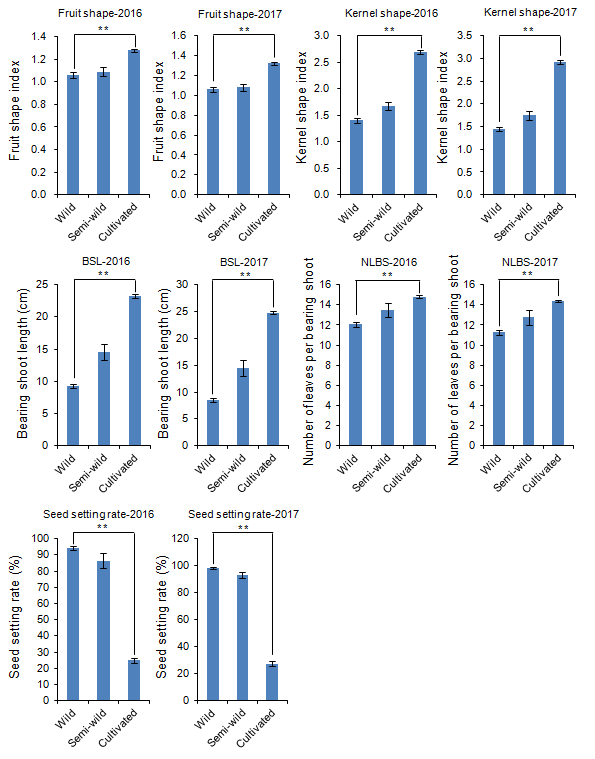


**Fig. S4 Comparative phenotypic analysis of wild, semi-wild and cultivated accessions.** Phenotypic data including fruit shape, kernel shape, BSL(bearing shoot length), NLBS (number of leaves per bearing shoot) and seed setting rate were collected in 2016 and 2017 years. Values are means ± SE. Differences between the wild and cultivatd accessions were analyzed by Welch’s *t* test. (**) indicated *P* < 0.01.


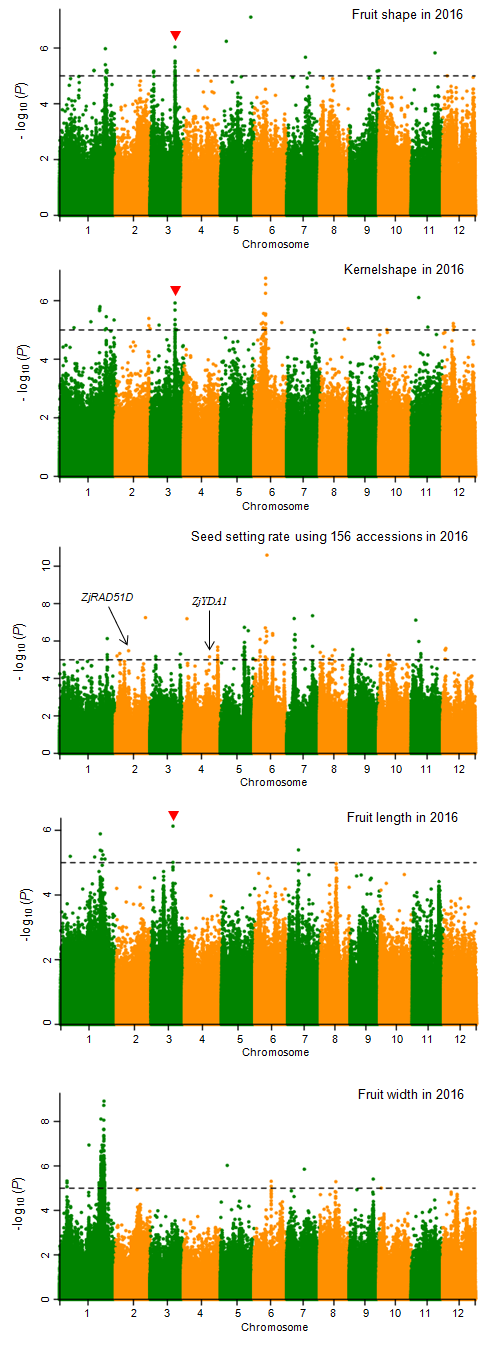


**Fig. S5 Genome-wide association studies of five horticultural traits.** Dashed lines represent significance thresholds (−log_10_*P* = 5). Red arrowheads indicate the position of peaks identified in this study.
